# 3D Medical Image Segmentation with 3D Modelling

**DOI:** 10.3390/bioengineering13020160

**Published:** 2026-01-29

**Authors:** Mária Ždímalová, Kristína Boratková, Viliam Sitár, Ľudovít Sebö, Viera Lehotská, Michal Trnka

**Affiliations:** 1 Department of Mathematics and Descriptive Geometry, Faculty of Civil Engineering, Slovak University of Technology in Bratislava, 810 05 Bratislava, Slovakia; kristina.boratkova@is.stuba.sk; 2 Faculty of Electrical Engineering and Information Technology, Slovak University of Technology in Bratislava, 810 05 Bratislava, Slovakia; xsitarv@stuba.sk; 3 2nd Department of Radiology, Faculty of Medicine, Comenius University in Bratislava, 812 50 Bratislava, Slovakia; ludovit.sebo@centrum.cz (Ľ.S.); viera.lehotska@ousa.sk (V.L.); 4 Institute of Medical Physics and Biophysics, Faculty of Medicine, Comenius University in Bratislava, 813 72 Bratislava, Slovakia

**Keywords:** 3D segmentation, image processing, Graphcut, DICOM data, tumor volumetry

## Abstract

**Background/Objectives:** The segmentation of three-dimensional radiological images constitutes a fundamental task in medical image processing for isolating tumors from complex datasets in computed tomography or magnetic resonance imaging. Precise visualization, volumetry, and treatment monitoring are enabled, which are critical for oncology diagnostics and planning. Volumetric analysis surpasses standard criteria by detecting subtle tumor changes, thereby aiding adaptive therapies. The objective of this study was to develop an enhanced, interactive Graphcut algorithm for 3D DICOM segmentation, specifically designed to improve boundary accuracy and 3D modeling of breast and brain tumors in datasets with heterogeneous tissue intensities. **Methods:** The standard Graphcut algorithm was augmented with a clustering mechanism (utilizing *k* = 2–5 clusters) to refine boundary detection in tissues with varying intensities. DICOM datasets were processed into 3D volumes using pixel spacing and slice thickness metadata. User-defined seeds were utilized for tumor and background initialization, constrained by bounding boxes. The method was implemented in Python 3.13 using the PyMaxflow library for graph optimization and pydicom for data transformation. **Results:** The proposed segmentation method outperformed standard thresholding and region growing techniques, demonstrating reduced noise sensitivity and improved boundary definition. An average Dice Similarity Coefficient (DSC) of 0.92 ± 0.07 was achieved for brain tumors and 0.90 ± 0.05 for breast tumors. These results were found to be comparable to state-of-the-art deep learning benchmarks (typically ranging from 0.84 to 0.95), achieved without the need for extensive pre-training. Boundary edge errors were reduced by a mean of 7.5% through the integration of clustering. Therapeutic changes were quantified accurately (e.g., a reduction from 22,106 mm^3^ to 14,270 mm^3^ post-treatment) with an average processing time of 12–15 s per stack. **Conclusions:** An efficient, precise 3D tumor segmentation tool suitable for diagnostics and planning is presented. This approach is demonstrated to be a robust, data-efficient alternative to deep learning, particularly advantageous in clinical settings where the large annotated datasets required for training neural networks are unavailable.

## 1. Introduction

The segmentation of three-dimensional radiological images constitutes a fundamental task in medical image processing, facilitating the isolation of specific anatomical or pathological structures from complex datasets acquired via modalities such as computed tomography (CT) or magnetic resonance imaging (MRI). This process is indispensable, as unsegmented images frequently exhibit overlapping tissues, which obscures target structures and complicates accurate diagnosis, treatment planning, and monitoring of disease progression. Visualization and quantitative analysis of regions of interest (ROIs), particularly tumors, are enhanced through the precise delineation of boundaries from surrounding tissues.

**Figure 1:**
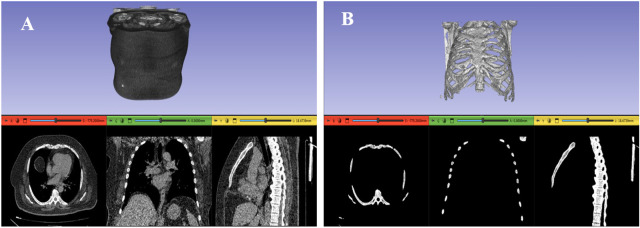
Comparison of 3D models without segmentation and after segmentation of bones [8,9,10,11]. (**A**) 3D model before segmentation. (**B**) 3D model after bone segmentation.

In oncology, the precise characterization of lesion morphology and extent is critical for clinical decision-making. Furthermore, segmentation underpins volumetrics, the quantitative assessment of tumor dimensions and volume, which is pivotal for the evaluation of therapeutic efficacy in interventions such as chemotherapy, radiotherapy, or radionuclide therapy. While the Response Evaluation Criteria in Solid Tumors (RECIST) employs unidimensional or bidimensional measurements, volumetric analysis provides a more comprehensive metric. Recent studies have demonstrated that multimodal imaging models, integrating techniques such as mammography and ultrasound, significantly enhance diagnostic specificity compared to single-modal approaches [1]. Such advanced volumetric data enable the detection of subtle changes in size, which may indicate early therapeutic success or resistance. Additionally, complementary techniques such as Particle-Induced X-ray Emission (PIXE) can enhance tumor characterization by analyzing trace elemental concentrations in segmented tissues [2,3,4,5,6]. Advanced segmentation techniques further refine the accuracy of these measurements [7]. Segmented images also facilitate the reconstruction of digital three-dimensional models, offering clinicians superior spatial insight compared to conventional two-dimensional slices [8]. As illustrated in Figure 1, a raw 3D model without segmentation (Figure 1A) provides limited medical utility due to tissue overlap, whereas a segmented model (Figure 1B) clearly delineated the structure of interest (e.g., the thoracic cage).

Image segmentation is defined as the partitioning of an image into constituent components (segments) based on shared characteristics such as intensity, color, or texture. In 3D medical imaging, this process extends to volumetric data, where individual points are treated as voxels with defined thickness and spatial coordinates. Common approaches include thresholding, region growing, and graph-based algorithms.

A comprehensive review of existing segmentation tools is crucial to establish the necessity of the proposed method. The fundamental distinction lies in the scope of utility: while general-purpose software provides flexible, broad toolsets, specific implementation, such as the graph cut method proposed herein, offer targeted algorithmic advantages to address inherent limitations in existing tools, such as the “shrinking bias” or memory consumption issues characteristic of large 3D datasets.

- **ImageJ/Fiji:** These open-source platforms are widely utilized for basic processing. Reliance is primarily placed on thresholding and region growing, which are computationally simple but prone to noise sensitivity. While there are plugins for advanced methods, core functions often lack global optimization guarantees.
- **ITK-SNAP:** This tool is renowned for its “snake” (active contour) evolution and manual painting features [12]. While effective for semi-automatic segmentation, active contours rely on local information and are susceptible to entrapment in local minima, thereby failing to reach a globally optimal solution.
- **3D Slicer:** A comprehensive platform that includes “GrowCut,” thresholding, and deep learning extensions [8,10]. Although GrowCut is efficient, specific regularization terms required for heterogeneous tumor boundaries may be lacking, potentially leading to less accurate boundaries than a custom, optimized graph cut implementation.

The proposed Graphcut method is distinguished by its targeting of specific shortcomings inherent in the aforementioned tools:

• **Global Optimality:** In contrast to “snakes” or region-growing methods that rely on local gradients, standard Graphcut approaches optimize a global energy function, providing consistent and reliable results.
• **Mitigation of “Shrinking Bias”:** A known limitation of standard Graphcuts is the bias toward producing smaller segments (shrinking bias), particularly for thin or elongated structures. The proposed method incorporates specific connectivity priors and clustering to mitigate this effect.
• **Data Constraints and Efficiency:** It is acknowledged that Deep Learning (DL) models currently represent the state-of-the art [13,14,15]. However, these models require large, annotated datasets (often exceeding 1000 samples) to facilitate effective training [13]. In clinical scenarios characterized by modest datasets and high variability across scanners, as is the case in this study, a robust, unsupervised, or semi-supervised energy minimization method like Graphcut often yields superior practical results without the risks associated with training on limited data.

Consequently, this study focuses on the development of an enhanced Graphcut algorithm for 3D DICOM segmentation, specifically augmented with clustering to improve boundary accuracy in breast and brain tumors. The aim is to demonstrate that for specific clinical datasets with limited training data, this optimized approach outperforms standard thresholding and region-growing techniques found in common software suites.

## 2. Materials and Methods

A semi-automatic 3D segmentation pipeline is proposed, integrating the Graphcut algorithm with a clustering-based intensity model to address the heterogeneity of tumor tissues. The workflow encompasses DICOM data acquisition, volumetric preprocessing, graph construction with specific capacity constraints, clustering-enhanced intensity modeling, and rigorous sensitivity analysis.

### 2.1. Data Acquisition and Preprocessing

Medical images were acquired in the DICOM standard [16]. To facilitate accurate segmentation, raw pixel data were transformed into a standardized intensity scale. For CT datasets, Hounsfield Unit (HU) transformation was applied using the linear rescale slope (*m*) and intercept (*b*) extracted from DICOM metadata (Equation (1)) [17]:

(1) HU= m·pixel_value+b

**Figure 2:**
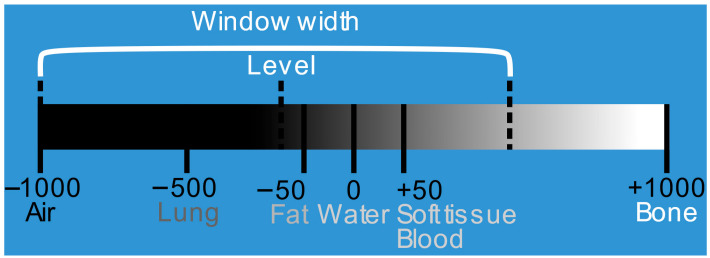
Hounsfield scale. Defining the window on the original scale.

This scale quantifies radiodensity, typically ranging from −1000 HU (air) to >1000 HU (bone), with water defined at 0 HU. Figure 2 illustrates this standard scale.

Subsequently, a Volume of Interest (VOI) windowing technique was employed to enhance contrast and focus on relevant tissues [18]. The intensity range was clipped based on the window *center* (*C*) and *width* (*W*) (Equation (2)) and normalized to an 8-bit integer range [0, 255] (Equation (3)) to optimize graph processing efficiency.

(2) $$I_{min}=C-\frac{W}{2},\;I_{max}=C+\frac{W}{2}$$

(3) $$I_{new}=\frac{I_{original}-I_{min}}{I_{max}-I_{min}}\;\cdot 255$$

**Figure 3:**
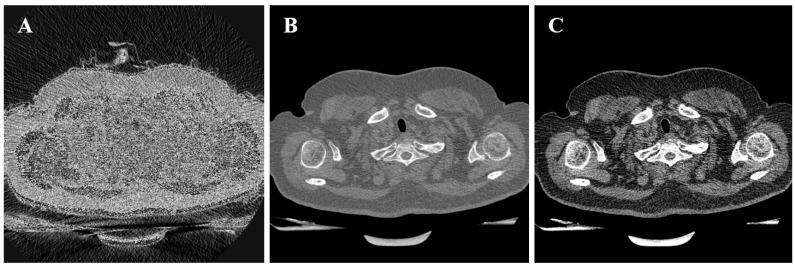
Comparison of transformation results for different views [9]. (**A**) Result of transformation without creating a window. (**B**) Result with a window size of 1000. (**C**) Result with a window size of 400, showing enhanced contrast.

For this study, a narrower window (e.g., width ≈ 400) was preferred over wide windows to enhance the visual distinction between tumor and surrounding soft tissue, as demonstrated in Figure 3.

### 2.2. Graph Construction and Energy Formulation

**Figure 4:**
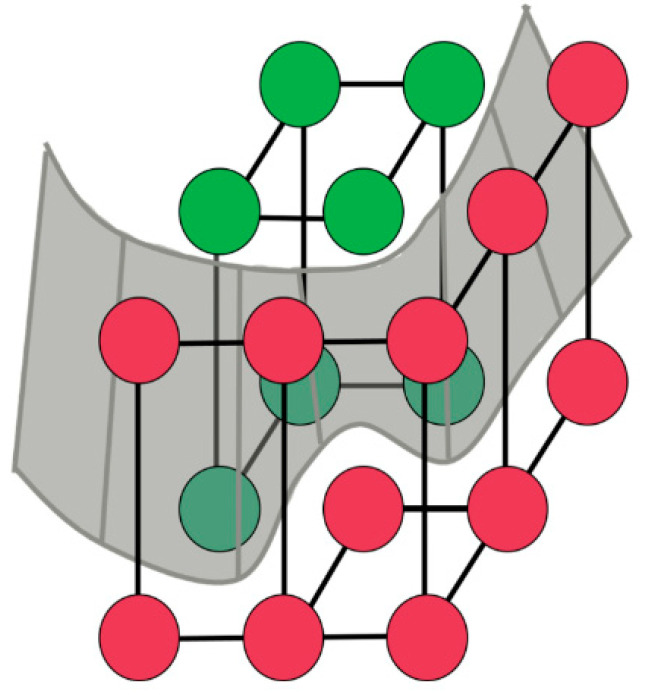
Illustration of minimum cut in 3D image segmentation, separating object nodes (*Source*) from background nodes (*Sink*). The red and green circles represent the seed points (or terminal nodes) used to initialize the 3D graph-cut segmentation algorithm. The green circles denote the Source nodes, which identify the object or foreground of interest. The red circles denote the Sink nodes, representing the background. These markers act as hard constraints, and the algorithm calculates the minimum cut (optimal boundary) that separates these two sets of nodes based on the image intensity gradients.

The 3D volumetric data is modeled as a directed graph $G=\;\langle V,\;E\rangle$, where *V* represents the set of voxels and *E* represents the set of edges. The edge set *E* consists N-links (neighborhood links), which connect adjacent voxels in a 6-connectivity 3D grid to ensure spatial continuity, and T-links (terminal links), which connect each voxel to two terminal nodes: the *Source* (*S*, object) and the *Sink* (*T*, background). The concept of the minimum cut separating these terminals in 3D space is illustrated in Figure 4.

The segmentation problem is formulated as a minimum cut problem minimizing the energy function *E*(*L*) [19,20]:

(4) $$E\left(L\right)=\;\sum_{p\in V}D_{p}\left(L_{p}\right)+\;\sum_{(p,\;q)\in N}V_{p,q}(L_{p},\;L_{q})$$

where *D_p_* denotes the data term derived from regional properties and *V_p,q_* denotes the smoothness term enforcing boundary continuity.

**Capacity definitions:** T-link capacities (*C_S_, C_T_*) were defined to enforce user-defined constraints. For voxels marked as Object seeds (*O*), capacities were set as *C_S_*(*p*) = *ϕ* · *K* and *C_T_*(*p*) = 0. Conversely, for Background seeds (*B*), capacities were set as *C_S_*(*p*) = 0 and *C_T_*(*p*) = *ϕ* · *K*. Here, *K* represents a sufficiently large constant to ensure seed adherence, and *ϕ* is an adjustable weighting parameter regulating the influence of seeds relative to boundary smoothness.

### 2.3. Clustering-Enhanced Intensity Modeling

**Figure 5:**
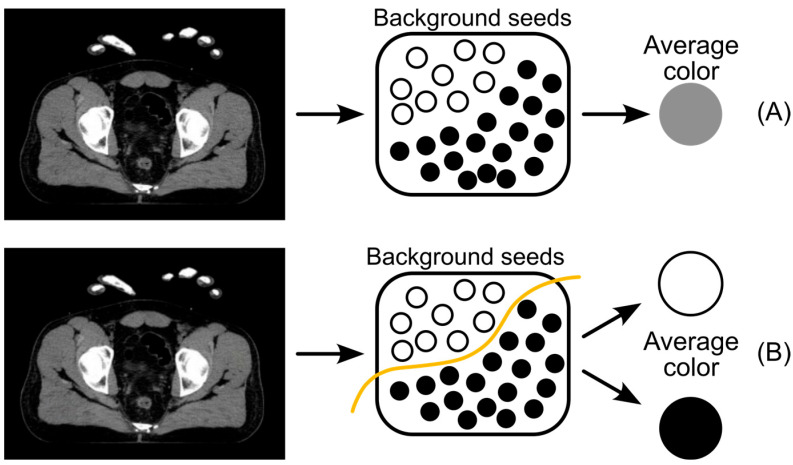
Schematic of the background modeling problem. (**A**) A standard single-mean model averages intensities, losing detail. (**B**) The proposed clustering model separates background seeds into multiple intensities classes (e.g., white bones vs. dark air). The black and white circles represent background seed points categorized into distinct intensity classes. White circles correspond to high-intensity structures (e.g., bones), while black circles correspond to low-intensity regions (e.g., air). By clustering these seeds into multiple classes (Model B), the algorithm better captures the heterogeneous nature of the background compared to a single-mean approach (Model A), leading to higher segmentation accuracy.

Standard Graphcut methods utilizing a single global intensity mean frequently fail to accurately segment tumors with heterogeneous intensities, such as those presenting with necrotic cores or enhancing rims, as illustrated in Figure 5.

To address this limitation, a *k*-means clustering approach (*k* = 2–5) was integrated into the intensity model [21]. Instead of a singular reference value, background and object seeds are clustered into *k* distinct groups with mean intensities {*m*_1_, …, *m_k_*}. For any unlabeled voxel with intensity *I_p_*, the background probability—and consequently the Sink capacity—is maximized based on the proximity to the nearest cluster center (Equation (5)). This multi-cluster modeling enables the simultaneous recognition of diverse tissue types (e.g., fat, muscle, bone) within the background, thereby significantly reducing segmentation errors at complex boundaries.

(5) $$C_{T}\left(p\right)\propto \;\underset{i=1\ldots k}{\max}\left(exp\left(\frac{{\left(I_{p}-\mu_{i}\right)}^{2}}{2\sigma^{2}}\right)\right)$$

### 2.4. Multi-Modal and Multi-Region Segmentation Capabilities

While primarily validated on CT and structural MRI, the proposed framework is designed to be modality-agnostic, supporting input from various imaging sequences including T1-weighted, T2-weighted, FLAIR, and Diffusion-Weighted Imaging (DWI) [22]. This flexibility is critical for characterizing the heterogeneous tumor microenvironment. Furthermore, the integration of the multi-label clustering model enables the method to distinguish and segment specific sub-regions of brain tumors, such as the peritumoral edema, necrotic core, and active enhancing tumor core, provided that appropriate seeds are initialized for each region. This capability aligns with the benchmarks established for multimodal brain tumor image segmentation [23].

### 2.5. Initialization and Boundary Constraints

**Figure 6:**
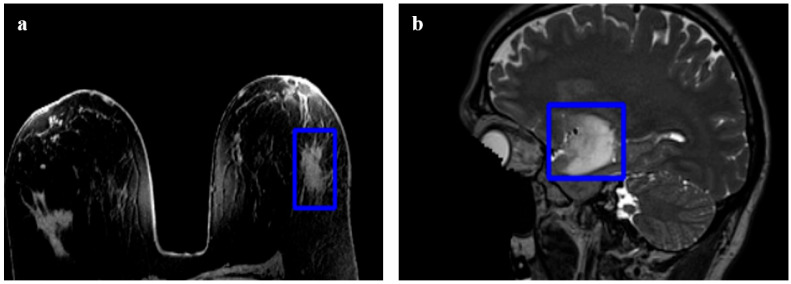
Examples of boundary constraints. (**a**) Bounding box around a breast tumor. (**b**) Bounding box around a brain tumor, excluding the skull.

A Bounding Box interaction model was implemented to restrict the search space and mitigate the shrinking bias inherent in graph-based methods [24]. By defining a 3D cuboid region of interest (ROI), computational resources are focused exclusively on the target volume, enhancing both processing efficiency and segmentation accuracy. Figure 6 demonstrates the application of bounding boxes to exclude irrelevant high-intensity structures (e.g., the skull when segmenting a brain tumor).

### 2.6. Implementation and Sensitivity Analysis

**Figure 7:**
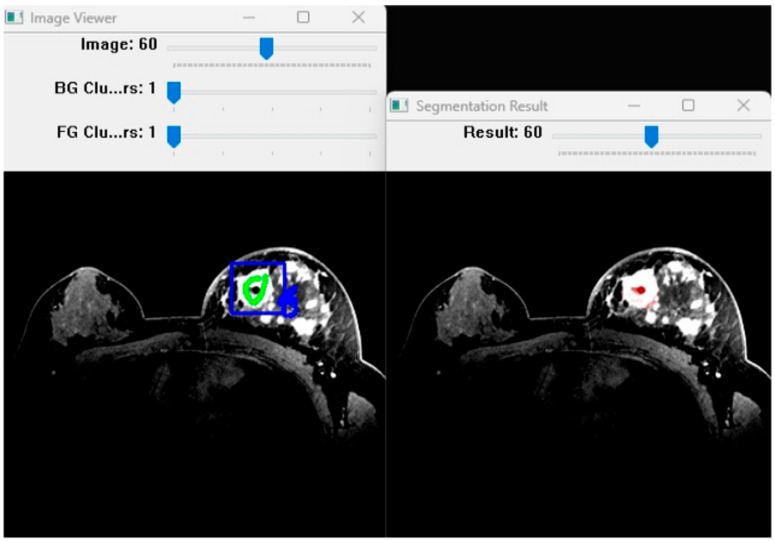
Software environment. The user interface allows adjustment of the cluster parameters (**top left**) and visualization of the segmentation result (**right**). The colors in the provided figures represent manual interaction markers (seeds) and the resulting segmentation calculated by the software. **Image 1 (3D Graph Representation):** The **green nodes** represent the object/foreground seeds (Source), while the **red nodes** represent the background seeds (Sink). These were manually marked within the software to define the terminal points for the graph-cut algorithm. The gray surface represents the calculated "minimum cut" or the final 3D boundary. **Image 2 (Background Modeling):** The **black and white circles** are representative background seeds sampled from different tissue types (e.g., white for bone, black for air). Model (B) illustrates how the software clusters these manual markers into distinct intensity classes to better handle background heterogeneity. **Image 3 (Software Interface):** This image shows the actual user interaction. The **green scribble** in the ‘Image Viewer’ represents the foreground seeds drawn by the user to mark the target lesion. The **blue scribble** represents the background seeds. In the ‘Segmentation Result’ window, the **red/white area** represents the final segmented object generated by the algorithm based on these manual inputs.

The algorithm was implemented in Python utilizing the PyMaxflow 1.3.2 library [25] for max-flow/min-cut optimization and pydicom 3.0.1 for DICOM data handling. Processing was executed on a workstation equipped with an Intel i7 CPU and 16 GB RAM. To evaluate the robustness of the method, a sensitivity analysis was conducted regarding the key parameters: the number of clusters (*k*) and the smoothness weight (*ϕ*). The parameter *k* was varied from 1 to 5 to assess its impact on boundary delineation in heterogeneous tissues. Additionally, inter-observer variability was assessed to estimate the uncertainty associated with manual seed initialization (Figure 7) [26,27].

## 3. Results

### 3.1. Evaluation Methodology and Gold Standard

To validate the accuracy of the proposed Graphcut method, the segmentation results were compared against a “Gold Standard” defined by manual segmentations performed by experienced radiologists using the 3D Slicer platform. For the breast tumor dataset, ground truth delineations provided by the RIDER Breast MRI dataset authors were utilized [28]. Quantitative performance was evaluated using three standard metrics:

- **Dice Similarity Coefficient (DSC):** This metric measures the spatial overlap accuracy between the prediction (*A*) and ground truth (*B*), defined as $DSC=\;\frac{2\left|A\cap B\right|}{\left|A\right|+\left|B\right|}$.
- **Intersection over Union (IoU):** Also known as the Jaccard Index, this is calculated as $IoU=\;\frac{\left|A\cap B\right|}{\left|A\cup B\right|}$
- **Pixel Accuracy:** This represents the ratio of correctly classified voxels to the total number of voxels.

### 3.2. Quantitative Performance

**Figure 8:**
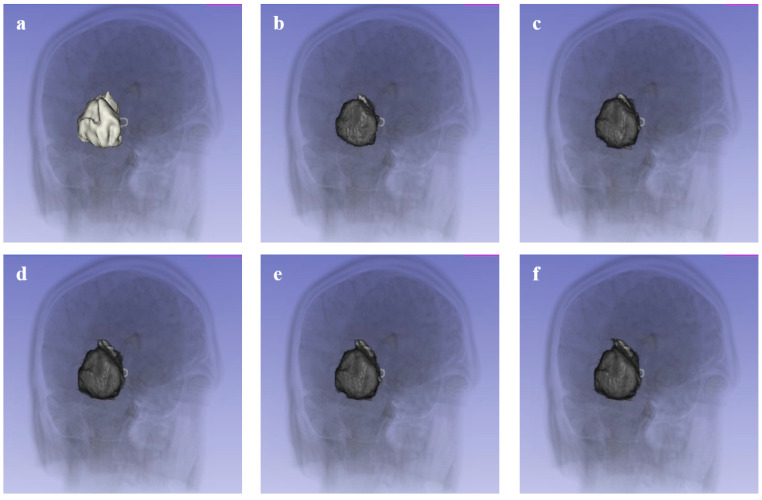
Impact of cluster count (*k*) on segmentation. (**a**) Manual ground truth. (**b**) Graphcut result (*k* = 1). (**c**–**f**) Graphcut results with increasing clusters (*k* = 2–5), showing tighter boundary adherence as *k* increases.

The performance of the proposed method on brain and breast tumor datasets is summarized in Table 1. High concordance with manual segmentations was achieved, with an average DSC exceeding 0.90 for both tumor types. Through the integration of clustering (using *k* = 3–4), boundary edge discrepancies were reduced by approximately 7.5% compared to the non-clustered Graphcut (*k* = 1), particularly in regions exhibiting gradient shading rather than sharp edges. The impact of cluster selection on segmentation granularity is illustrated in Figure 8.

### 3.3. Comparison with Standard Methods

The proposed method was compared against standard Thresholding and Region Growing techniques available in 3D Slicer.

**Figure 9:**
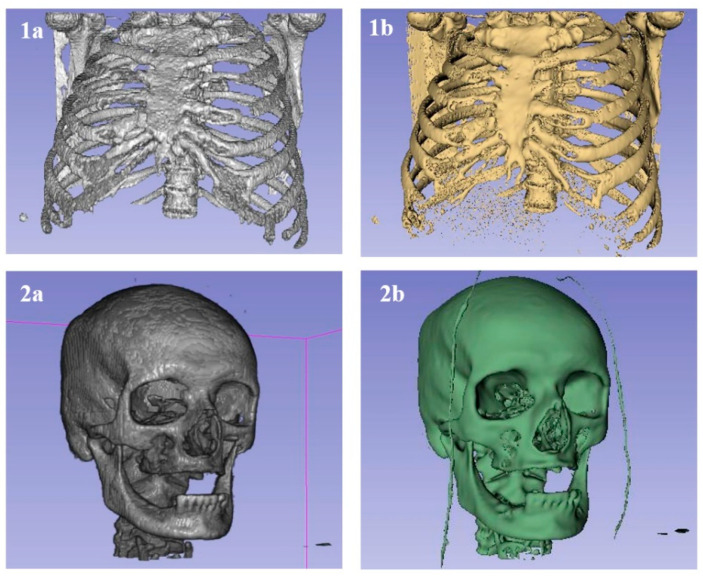
Comparison with Thresholding. (**1a**) Rib segmentation using Graphcut showing clean structure. (**1b**) Thresholding result showing significant noise. (**2a**) Skull segmentation using Graphcut. (**2b**) Thresholding result showing artifacts.

**Figure 10:**
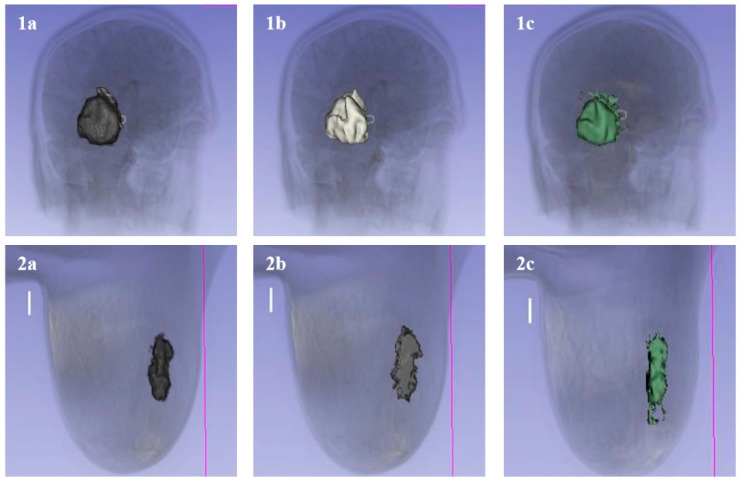
Comparison of segmentation methods on brain (**Top**) and breast (**Bottom**) tumors. (**a**) Proposed Graphcut method. (**b**) Manual Segmentation (Gold Standard). (**c**) Region Growing method, showing leakage/under-segmentation compared to the proposed method. The lines visible in the 3D reconstructions represent the computed 3D boundaries or ‘outlines’ of the segmented structures. In the context of the graph-cut algorithm, these lines illustrate the finalized ‘minimum cut’ surface where the algorithm has separated the object nodes from the background. They serve as a visual verification of the spatial extent and surface continuity of the segmented anatomical model (such as the skull or rib cage) within the 3D volume.

**Figure 11:**
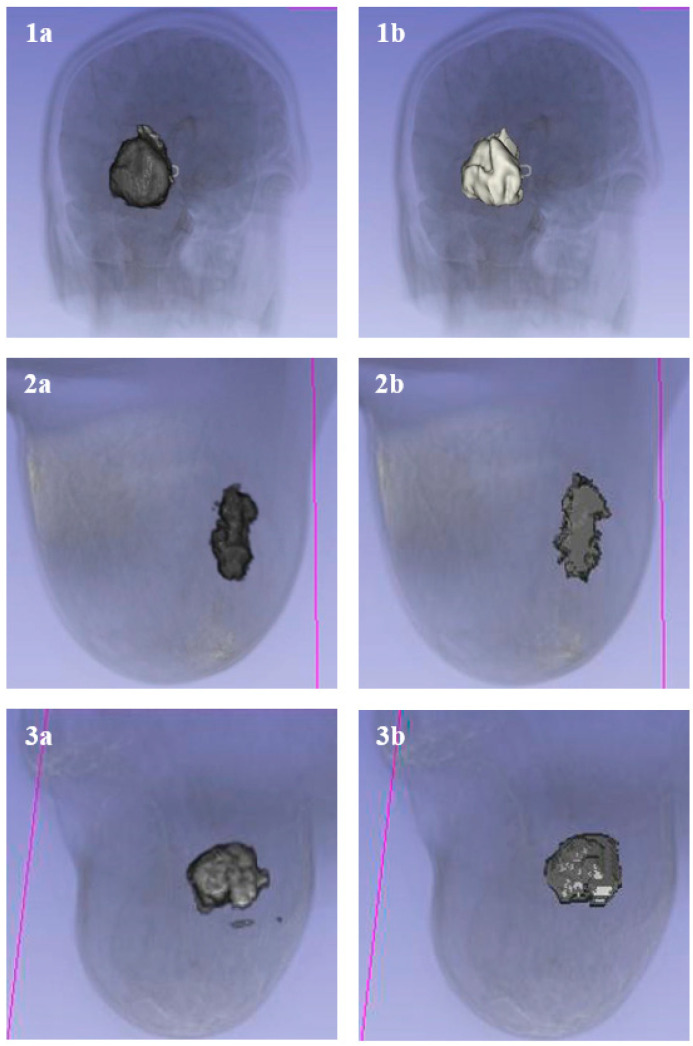
3D model comparison. (**a**) Resulting tumor segmentation using proposed Graphcut method. (**b**) Manual tumor segmentation (Gold Standard). The proposed method recovers the complex 3D shape with high fidelity. The lines visible in the 3D reconstructions represent the computed 3D boundaries or ‘outlines’ of the segmented structures. In the context of the graph-cut algorithm, these lines illustrate the finalized ‘minimum cut’ surface where the algorithm has separated the object nodes from the background. They serve as a visual verification of the spatial extent and surface continuity of the segmented anatomical model (such as the skull or rib cage) within the 3D volume.

- **vs. Thresholding:** Thresholding alone failed to distinguish tumors from surrounding tissues of similar intensity (e.g., distinguishing bone from contrast-enhanced vessels). Significant noise and artifacts were observed in thresholding results (see Figure 9), whereas the Graphcut method effectively suppressed this noise through global optimization and smoothness constraints.

- **vs. Region Growing:** While better localization was offered by region growing compared to thresholding, “leakage” into adjacent healthy tissues was frequently observed where boundaries were weak. Tighter boundary adherence was maintained by the proposed method, which was reinforced by the clustering model and bounding box, thereby matching the manual ground truth more closely (Figure 10 and Figure 11).

### 3.4. Comparison with Deep Learning Benchmarks

To evaluate the performance of the proposed clustering-enhanced Graphcut, results were compared with established deep learning benchmarks, such as the standard 3D U-Net and transformer-based models. Meta-analyses of international competitions, specifically the Multimodal Brain Tumor Segmentation (BraTS) challenges, indicate that state-of-the-art automated methods typically achieve Dice Similarity Coefficients (DSCs) for the whole tumor in the range of 0.84 to 0.95. For instance, recent studies employing deep learning for brain tumor segmentation with minimized MRI data reported DSC values of 0.867 for the enhancing tumor and 0.926 for the tumor core [29]. Similarly, AI-based systems for breast cancer detection using Convolutional Neural Networks (CNNs) like ResNet-18 have demonstrated high accuracy in classification tasks [30].

The proposed method achieved a comparable average DSC of 0.92 ± 0.07 for brain tumors and 0.90 ± 0.05 for breast tumors, placing it within the performance envelope of modern high-end algorithms. The primary distinction lies in the operational requirements: while deep learning architectures require extensive pre-training on thousands of manually annotated slices to generalize, competitive results are achieved by the proposed approach in a zero-shot manner without the need for high-end GPU hardware.

### 3.5. Sensitivity and Variability Analysis

The sensitivity of the segmentation to the clustering parameter *k* and the smoothness weight *ϕ* was analyzed.

- **Parameter Sensitivity:** Varying *k* from 2 to 5 resulted in minor fluctuations in DSC (<±0.03), indicating that the method is relatively robust to the choice of *k*, provided that *k* ≥ 2 to capture background heterogeneity. This aligns with findings that seeded segmentation frameworks are generally stable against local parameter perturbations [26].
- **Inter-Observer Variability:** The dependence on manual seed initialization introduces potential inter-observer variability. However, the standard deviations reported in Table 1 (±0.05 to ±0.07) suggest that this variability remains within clinically acceptable limits. This consistency is critical for reliable uncertainty estimation in medical image segmentation [27].

### 3.6. Volumetric Analysis

**Figure 12:**
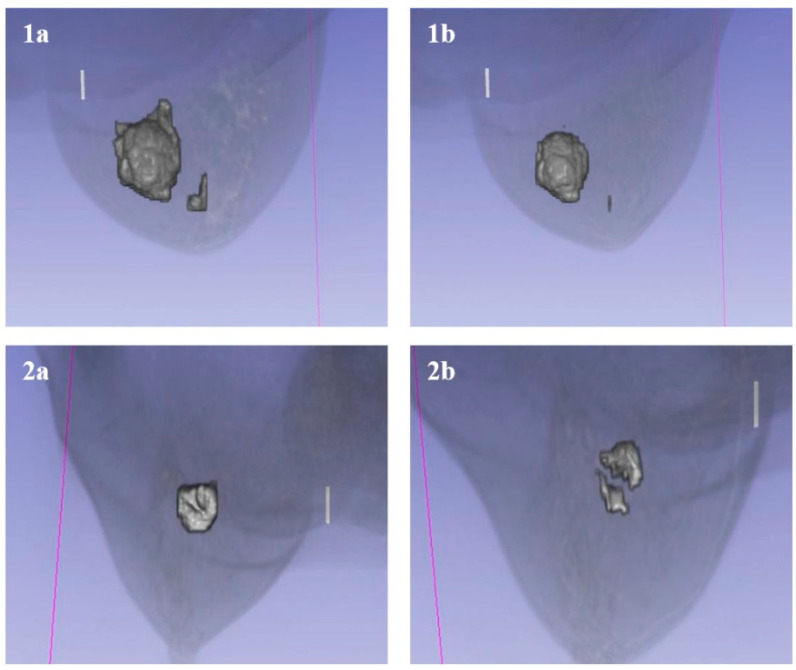
Volumetric analysis of tumor response to treatment. (**1a**,**2a**) Pre-treatment tumor volumes. (**1b**,**2b**) Post-treatment tumor volumes, showing significant regression quantified by the software.

High utility for volumetric monitoring, a critical requirement for RECIST-based therapy evaluation, was demonstrated. In a representative case study of tumor response to treatment, a significant reduction in tumor volume was quantified across two timepoints (Figure 12):

• **Timepoint 1:** 22,106.02 mm^3^
• **Timepoint 2:** 14,270.05 mm^3^

This precise quantification (<15 s processing time per volume) supports the application of this tool for rapid evaluation in clinical workflows.

## 4. Discussion and Conclusions

### 4.1. Comparison with Existing Segmentation Approaches

A specialized 3D Graphcut implementation is presented, addressing specific limitations inherent in general-purpose segmentation tools. While software such as ITK-SNAP and 3D Slicer offer robust semi-automatic tools (e.g., active contours, region growing), reliance is often placed on local gradient information, rendering them susceptible to entrapment in local minima [12]. In contrast, the global optimization framework of Graph Cuts is utilized in this method, ensuring a mathematically optimal segmentation for the defined energy function. A critical advancement is the integration of *k*-means clustering into the energy function. Standard Graphcut methods are frequently hindered by shrinking bias (preference for shorter boundaries) and the assumption of homogeneous object intensities. By clustering background and foreground seeds into multiple intensity classes (*k* = 2–5), tumors with heterogeneous appearances, such as those exhibiting necrotic cores or enhancing rims, are successfully delineated, whereas standard thresholding or simple Graphcut implementations frequently result in misclassification.

### 4.2. Comparative Analysis with Deep Learning Frameworks

While the quantitative results reported in Section 3.4. confirm that the proposed Graphcut method achieves segmentation accuracy comparable to state-of-the-art Deep Learning (DL) benchmarks [29,30], the primary distinction lies in the operational paradigm rather than raw metric performance. Deep learning models, such as U-Net and transformer-based architectures, have established themselves as the gold standard for high-throughput analysis [13,14]. However, this performance is predicated on the availability of massive, annotated training datasets. In the context of this study, the comparable Dice Similarity Coefficients (DSC ≈ 0.92) demonstrate that an explicitly defined energy-minimization framework can rival the performance of data-hungry neural networks in specific, low-resource clinical scenarios.

The proposed method addresses the critical “data scarcity” bottleneck often encountered in rare tumor analysis or single-institution studies. Unlike deep learning architectures, which require extensive pre-training to generalize, the clustering-enhanced Graphcut operates in a zero-shot manner, ensuring consistent performance independent of training set size. Furthermore, the “black box” nature of neural networks often obscures the decision-making process, raising concerns regarding explainability in diagnostic tasks. Conversely, the proposed method offers full mathematical interpretability: the segmentation boundary is explicitly defined by the global minimum of the energy function *E*(*L*). This transparency allows clinicians to verify that the segmentation is driven by specific intensity gradients and spatial coherence rather than opaque learned features.

Despite these advantages, the superiority of deep learning in specific operational contexts must be recognized. The most significant limitation of the proposed interactive framework, compared to DL, is the requirement for manual initialization (seeds and bounding boxes). In high-volume clinical workflows processing hundreds of scans daily, the fully automated nature of AI models—which eliminates human intervention and inter-observer variability—remains a distinct advantage. While the Graphcut method excels in precision and interpretability for individual, complex cases, deep learning remains the optimal solution for large-scale, automated screening pipelines where high-performance computing infrastructure is available.

### 4.3. Methodological Robustness and Sensitivity

The robustness of the proposed framework was corroborated through sensitivity analysis. The variation in the clustering parameter *k* (2 ≤ *k* ≤ 5) demonstrated minimal impact on global segmentation accuracy, suggesting that the method is stable provided that sufficient clusters are initialized to capture tissue heterogeneity [26]. Although manual seed initialization introduces a degree of inter-observer variability, the observed standard deviations in DSC (±0.07) indicate that this variability is contained within limits comparable to those observed in other semi-automatic frameworks [27]. This stability is essential for clinical reproducibility.

### 4.4. Clinical Implications and Volumetry

**Figure 13:**
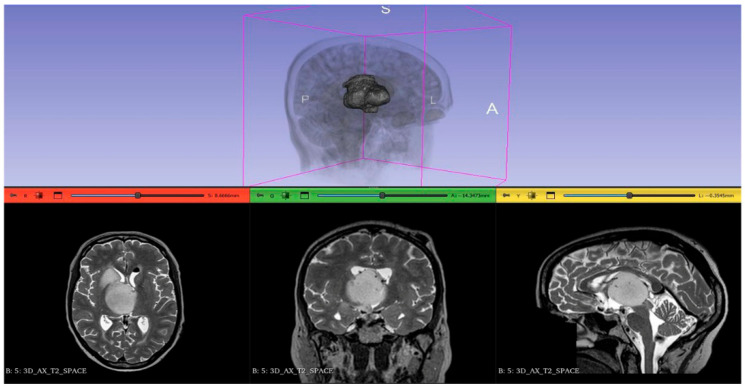
3D visualization of a segmented brain tumor using the proposed pipeline. (**Top**) 3D surface model rendering. (**Bottom**) Multi-planar reconstruction showing the tumor extent in axial, coronal, and sagittal views.

The ability to accurately quantify tumor volume has direct clinical implications. As demonstrated in results, the method successfully tracked tumor regression (e.g., from ~22,000 mm^3^ to ~14,000 mm^3^) across treatment timepoints. This volumetric sensitivity surpasses unidimensional RECIST measurements, potentially enabling earlier detection of treatment non-response. Furthermore, the segmentation facilitates the generation of interactive 3D models, as shown in Figure 13, which allows clinicians to visualize the tumor’s spatial orientation relative to critical anatomical structures (e.g., ventricles in the brain) for surgical planning.

### 4.5. Limitations and Future Directions

Certain limitations of this study must be acknowledged. The validation was conducted on a modest dataset; consequently, the generalizability of the method across a broader spectrum of acquisition protocols and scanner manufacturers remains to be fully quantified. Future validation efforts will prioritize cross-dataset experiments and multi-institutional cross-validation to rigorously assess performance stability [29].

Despite the distinct advantages of the proposed method in data-scarce environments, areas where deep learning architectures maintain superiority must be recognized. The most significant advantage of DL-based models is their fully automated nature, which eliminates the need for manual seed placement or bounding box initialization. In high-throughput clinical settings where hundreds of scans are processed daily, the capability to perform segmentation with zero human intervention is a critical factor for operational efficiency. Furthermore, while the clustering-enhanced Graphcut relies primarily on intensity gradients and local voxel relationships, AI models possess the capacity to distinguish between tissues with identical intensities by recognizing global spatial context and anatomical location. Finally, the elimination of manual interaction in DL models removes inter-observer variability, whereas Graphcut results may vary slightly based on seed placement, a trained AI model provides a perfectly reproducible output for identical input data. Consequently, deep learning remains the gold standard for large-scale, automated diagnostic pipelines where high-performance computing infrastructure is available.

### 4.6. Conclusions

An enhanced 3D Graphcut segmentation tool tailored for medical DICOM datasets has been developed and validated. Through the incorporation of clustering to address tissue heterogeneity and the optimization of the pipeline for standard hardware, a precise, efficient, and accessible solution for tumor volumetry is offered. This method effectively bridges the gap between manual delineation and fully automated deep learning, providing clinicians with a reliable tool for diagnostics and treatment monitoring in settings where large-scale training data is unavailable.

## Figures and Tables

**Table 1:** Average quantitative metrics (±standard deviation) for Graphcut 3D segmentation performance on brain and breast tumors.

Metric	Brain Tumors	Breast Tumors	Notes
IoU	0.86 ± 0.06	0.84 ± 0.07	Enhanced by bounding box constraints.
DSC	0.92 ± 0.07	0.90 ± 0.05	Clustering reduced boundary errors by ~5–10%.
Pixel Accuracy	0.95 ± 0.03	0.93 ± 0.04	Outperforms thresholding by minimizing noise.
Processing Time (s/3D stack)	15 ± 3	12 ± 2	3D stacks approx. ~100 slices (256 × 256 resolution).

## Data Availability

The raw data supporting the conclusions of this article are available from public repositories (The Cancer Imaging Archive) as cited in the references [28,31]. Processed data will be made available by the authors on request.

## Abbreviations

CT	Computed Tomography
MRI	Magnetic Resonance Imaging
ROI	Region of Interest
RECIST	The Response Evaluation Criteria In Solid Tumors
PIXE	Particle-Induced X-ray Emission
HU	Hounsfield Unit
VOI	Volume of Interest
GUI	Graphical User Interface
DWI	Diffusion-Weighted Imaging
IoU	Intersection over Union
DSC	Dice Similarity Coefficient
CNN	Convolutional Neural Network
